# A novel autoantibody against fibronectin leucine-rich transmembrane protein 2 expressed on the endothelial cell surface identified by retroviral vector system in systemic lupus erythematosus

**DOI:** 10.1186/ar3897

**Published:** 2012-07-02

**Authors:** Tsuyoshi Shirai, Hiroshi Fujii, Masao Ono, Kyohei Nakamura, Ryu Watanabe, Yumi Tajima, Naruhiko Takasawa, Tomonori Ishii, Hideo Harigae

**Affiliations:** 1 Department of Hematology and Rheumatology, Tohoku University Graduate School of Medicine, 1-1 Seiryo-cho, Aoba-ku, Sendai, Miyagi 980-8574, Japan; 2 Department of Histopathology, Tohoku University Graduate School of Medicine, 1-1 Seiryo-cho, Aoba-ku, Sendai, Miyagi 980-8574, Japan

## Abstract

**Introduction:**

Anti-endothelial cell antibodies (AECAs) are thought to be critical for vasculitides in collagen diseases, but most were directed against molecules localized within the cell and not expressed on the cell surface. To clarify the pathogenic roles of AECAs, we constructed a retroviral vector system for identification of autoantigens expressed on the endothelial cell surface.

**Methods:**

AECA activity in sera from patients with collagen diseases was measured with flow cytometry by using human umbilical vein endothelial cells (HUVECs). A cDNA library of HUVECs was retrovirally transfected into a rat myeloma cell line, from which AECA-positive clones were sorted with flow cytometry. cDNA of the cells was analyzed to identify an autoantigen, and then the clinical characteristics and the functional significance of the autoantibody were evaluated.

**Results:**

Two distinct AECA-positive clones were isolated by using serum immunoglobulin G (IgG) from a patient with systemic lupus erythematosus (SLE). Both clones were identical to cDNA of fibronectin leucine-rich transmembrane protein 2 (*FLRT2*). HUVECs expressed FLRT2 and the prototype AECA IgG bound specifically to *FLRT2*-transfected cells. Anti-FLRT2 antibody activity accounted for 21.4% of AECAs in SLE. Furthermore, anti-FLRT2 antibody induced complement-dependent cytotoxicity against FLRT2-expressing cells.

**Conclusions:**

We identified the membrane protein FLRT2 as a novel autoantigen of AECAs in SLE patients by using the retroviral vector system. Anti-FLRT2 antibody has the potential to induce direct endothelial cell cytotoxicity in about 10% of SLE patients and could be a novel molecular target for intervention. Identification of such a cell-surface target for AECAs may reveal a comprehensive mechanism of vascular injury in collagen diseases.

## Introduction

Vascular endothelial cells (ECs) represent the boundary between blood and tissue, and contribute to the process of inflammation. Anti-endothelial cell antibodies (AECAs) were first described in 1971 and defined as autoantibodies that target antigens present on the EC membrane [1,2]. AECAs have been detected in a number of patients with collagen diseases, including systemic lupus erythematosus (SLE), and were shown to be correlated to disease activity [3,4]. SLE is one of the diseases in which AECAs are frequently detected, and they are considered to play a role in the pathogenesis, especially in lupus nephritis [3,4]. In addition, SLE patients have an increased risk of cardiovascular disease originating from SLE itself, and it has been reported that AECAs play roles in atherosclerotic events [5].

AECAs have the potential to induce vascular lesions directly because their targets are expressed on ECs, which are always in contact with these circulating antibodies. AECAs are considered to play roles in the development of pathologic lesions by EC cytotoxicity (complement-dependent cytotoxicity (CDC) and antibody-dependent cell-mediated cytotoxicity (ADCC)), activation of EC (proinflammatory cytokine secretion and expression of adhesion molecules), induction of coagulation, and induction of apoptosis [6-9].

Although new biologic drugs have been applied to the treatment of SLE, currently available therapies often introduce the additional risk of immunosuppression [10]. Bloom *et al. *[11] proposed a model for customized and specific therapeutic approaches against a highly pathogenic subset of lupus antibodies by using small molecules that neutralize them. AECAs may be good targets for such interventions, and identification of cell-surface targets of AECAs is required.

Target antigens of AECAs had been investigated intensively, but they are heterogeneous and classified into the following three groups: membrane component, ligand-receptor complex, and molecule adhering to the plasma membrane [12]. The cellular localization of the target antigen is considered to be a critical factor in the pathogenesis of autoantibodies [13], and it is generally accepted that autoantibodies against integral membrane proteins are usually pathogenic [14]. Although AECAs must be directed against the cell surface, most of the molecules reported to date as targets for AECAs are intracellular proteins [2,4,6,15]. Several groups have recently identified targets of AECAs by proteomics analysis [16,17]. However, extraction of some membrane proteins is difficult in proteomics analysis, and this may be one of the reasons that such proteins were not identified as AECA targets [2].

We constructed a retroviral vector system [18] to identify autoantigens expressed on the EC surface by using flow cytometry and identified the membrane protein fibronectin leucine-rich transmembrane protein 2 (FLRT2) as a novel autoantigen of AECAs in patients with SLE.

## Materials and methods

### Sources of human sera

Two hundred thirty-three patients with collagen diseases (196 female and 37 male patients) were enrolled in the study. The mean age was 42.5 years, with a range of 18 to 72 years. The patients comprised 95 with SLE and 138 with other collagen diseases. All of the patients were diagnosed according to the respective classification criteria [19-32]. Thirty-five age- and sex-matched healthy donors were enrolled as a control group. Sera were collected and stored at -20°C until use. All subjects gave written consent after the purpose and potential risks involved in the study were explained. The study protocol complied with the principles of the Declaration of Helsinki and was approved by the Ethical Committee of Tohoku University Graduate School of Medicine.

### Cell culture

Human umbilical vein endothelial cells (HUVECs), human aortic endothelial cells (HAECs), human lung microvascular endothelial cells (HMVEC-Ls), and EGM-2 medium were purchased from Lonza (Basel, Switzerland). Human renal glomerular endothelial cells (HRGECs) and endothelial cell medium were purchased from ScienCell Research Laboratories (Carlsbad, CA, USA). The cells were grown in 5% CO_2 _at 37°C on polystyrene flasks (BD Biosciences, Bedford, MA, USA). These ECs were used at sooner than the fifth passage. HEK293T cells were purchased from American Type Culture Collection (ATCC) (Manassas, VA, USA), Plat-E and Plat-GP packaging cells were purchased from Cell Biolabs (San Diego, CA, USA) and cultured in Dulbecco modified Eagle medium (DMEM) (Sigma, St. Louis, MO, USA) supplemented with 10% fetal bovine serum (FBS) (HyClone, Logan, UT, USA). Rat myeloma cells, YB2/0, were purchased from ATCC and cultured in RPMI1640 medium (Sigma) containing 10% FBS.

### IgG purification

IgG fractions were purified from sera by using HiTRAP Protein G HP columns (Amersham Biosciences, Roosendaal, The Netherlands). The concentration of purified IgG was determined by measuring the OD at 280 nm (OD_280_). Purified IgG was stored at -20°C until use.

### Flow cytometry

Binding activities of antibodies to the surface of ECs and FLRT2 molecules were measured by using FACSCalibur and FACSCanto II (Becton Dickinson, Franklin Lakes, NJ, USA) [17], and the data were analyzed with FlowJo Software (Tree Star, Ashland, OR, USA). In brief, attached cells were dissociated from plates by using Cell Dissociation Solution (Sigma) and washed with phosphate-buffered saline (PBS). Aliquots of 1 × 10^5 ^cells/tube were incubated in blocking buffer (PBS containing 1% bovine serum albumin and 50 mg/ml goat gamma globulin fraction (Sigma)) with primary antibodies at 4°C for 30 minutes. After washing, cells were incubated with secondary antibodies and 7-amino-actinomycin D (7-AAD) (BD Biosciences) at 4°C for 30 minutes and analyzed with flow cytometry.

Primary antibodies included 1:10 diluted human serum, 0.5 mg/ml of purified human IgG, and 10 μg/ml goat anti-human FLRT1/FLRT2/FLRT3 antibody (R&D Systems, Minneapolis, MN, USA). Secondary antibodies included 1:50 diluted fluorescein isothiocyanate (FITC) or phycoerythrin (PE)-conjugated goat anti-human IgG (Abcam, Cambridge, UK), PE-conjugated donkey anti-goat IgG (Abcam), PE-conjugated mouse anti-human IgG1/IgG2/IgG3/IgG4 antibody (Beckman Coulter, Fullerton, CA, USA), and DyLight 650-conjugated anti human IgM antibody (Abcam). For staining of the intracellular FLRT2 domain, IntraStain (Dako, Glostrup, Denmark) and anti-human FLRT2 antibody (K-20) (Santa Cruz Biotechnology, Santa Cruz, CA, USA) were used.

For measurement of AECA activity, the relative mean fluorescence intensity (MFI) ratio was calculated as follows: (sample MFI - control MFI)/control MFI × 100 [33]. Relative MFI ratio of mean + 3 standard deviations (SD) among the control group was defined as the cutoff value for AECAs. For measurement of anti-FLRT2 activity against the cell-surface domain, the relative MFI ratio was calculated as follows: (MFI against FLRT2-expressing cells - MFI against non-FLRT2-expressing cells)/MFI against non-FLRT2-expressing cells × 100. In each set of experiments, relative MFI ratios of titrated reference serum with high anti-FLRT2 activity were calculated, and a standard curve was generated. The relative MFI ratio was converted to arbitrary units (AUs) according to the standard curve. AU of mean + 3 SD in the control group was defined as the cutoff value for the anti-FLRT2 antibody. Recombinant human FLRT2 (R&D Systems) was added at the indicated dose in inhibition tests. The percentage inhibition was calculated as follows: % inhibition = (AECA titer of sample serum - AECA titer of sample serum with inhibitor)/AECA titer of sample serum × 100.

### HUVEC cDNA library

Total RNA was generated from HUVECs by using an RNeasy Mini Kit (Qiagen, Hilden, Germany), and poly(A)+ RNA was purified with an mRNA Purification Kit (GE Healthcare, Little Chalfont, Buckinghamshire, UK). Double-stranded cDNA was synthesized by using a cDNA library construction kit (Takara Bio, Shiga, Japan). DNA fragments > 1,000 bp in length were ligated into the pMX vector (kindly donated by Toshio Kitamura, University of Tokyo, Tokyo, Japan).

### Screening of cDNA library

The HUVEC cDNA library in pMX was retrovirally transfected into the YB2/0 rat myeloma cell line [34]. Aliquots of 1 × 10^7 ^YB2/0 cells expressing the HUVEC cDNA library were incubated with 0.5 mg/ml of IgG with high AECA activity at 4°C for 30 minutes. After washing, cells were incubated with FITC-conjugated goat anti-human IgG and 7-AAD at 4°C for 30 minutes. The cells showing a high level of FITC fluorescence signal were sorted with FACS Vantage (Becton Dickinson). Sorted cells were kept in culture until the cell number increased sufficiently for the next round of sorting. Subcloning of cells bound to IgG with AECA activity was performed by the limiting dilution method.

Genomic DNAs of clones were purified by using the Wizard SV Genomic DNA Purification system (Promega Corporation, Madison, WI, USA). DNA fragments from the HUVEC cDNA library were amplified by polymerase chain reaction (PCR) by using TaKaRa LA Taq (Takara Bio) with primers corresponding to the 5' and 3' ends of the multiple cloning site of pMX (5'-GGTGGACCATCCTCTAGACTG, 3'-CCTTTTTCTGGAGACTAAAT, respectively). The PCR products were cloned into the pCR-TOPO vector (Invitrogen), and DNA sequences were analyzed with the BLAST program.

### Expression of FLRT2 in HEK293T cells

The full-length FLRT2 fragment was amplified by PCR from genomic DNA of FLRT2-expressing YB2/0 clone sorted as described earlier, by using Phusion High-Fidelity DNA Polymerase (Finnzymes, Keilaranta, Espoo, Finland). Primer sequences were as follows: 5'-CCCACCACATTGTATTTTATTTCC, 3'-CTTGATAACGCTGGGCCTCT. The FLRT2 fragment was inserted into the pMX-IRES-GFP vector (Cell Biolabs). An FLRT2 expression vector with deletion of the unique region was made by using an In-Fusion HD Cloning Kit (Clontech Laboratories, Madison, WI, USA) with two PCR segments constructed to omit the unique region (363 to 419 amino acids) and inserted into the pMX-IRES-GFP vector. pMX-FLRT2-IRES-GFP was transfected directly into HEK293T cells with FuGENE HD (Roche Diagnostics, Basel, Switzerland) or retrovirally transfected into HEK293T cells. Full-length FLRT1 and FLRT3 fragments were amplified as described earlier and inserted into the pMX-IRES-GFP vector.

### Western blotting

Cells were lysed in RIPA buffer (Cell Signaling Technology, Danvers, MA, USA). The lysate was mixed with 5 × sodium dodecyl sulfate (SDS) sample buffer and separated by electrophoresis on an 8% polyacrylamide gel. The proteins were then transferred onto Immobilon Transfer Membranes (Millipore, Billerica, MA, USA). The membranes were treated with 0.1 μg/ml of goat anti-FLRT2 antibody and IRDye680-conjugated donkey anti-goat IgG (LI-COR Biosciences, Lincoln, NE, USA), and fluorescence intensity was determined with the Odyssey Infrared Imaging System (LI-COR).

### CDC

CDC was assessed by the tetrazolium salt reduction method by using WST-1 (Roche Diagnostics) [35-37]. In brief, cells were seeded in 96-well culture plates at a concentration of 4 × 10^4 ^cells per well and cultured overnight. Cells were incubated with 100 μl of diluted IgG for 30 minutes followed by addition of 50 μl of rabbit complement (Cedarlane Laboratories, Burlington, ON, Canada) at the indicated concentrations for 2 hours at 37°C. Then 15 μl of WST-1 was added, and cells were incubated for an additional 4 hours. Absorbance at 450 nm (A_450_) was measured and expressed as relative fluorescence units (RFUs), reflecting the number of viable cells. Triton X-100 (1%) and heat-inactivated complement were added to the wells to measure background or maximal absorbance of WST-1, respectively. Recombinant FLRT2 was added in the inhibition tests. The percentage cytotoxicity for each sample was calculated by using the formula:

% cytotoxicity=(maximal RFU-sample RFU)/(maximal RFU-background RFU)×100.

### ADCC

ADCC was determined by using the LDH Cytotoxicity Detection Kit (Takara Bio) and the manufacturer's protocol [36].

The percentage cytotoxicity was calculated as follows:

% cytotoxicity=(experimental-effector spontaneous-target spontaneous)/(target maximum-target spontaneous)×100.

### Detection of adhesion molecule expression

HUVECs were cultured overnight in 96-well culture plates and incubated with IgG (640 μg/ml) for 6 hours at 37°C. Harvested cells were stained with PE-conjugated anti-CD62E antibody (BioLegend, San Diego, CA, USA), allophycocyanin (APC)-conjugated anti-CD106 antibody (BioLegend), and Pacific blue-conjugated anti-CD54 antibody (BioLegend), and were analyzed with flow cytometry.

### Detection of EC apoptosis

HUVECs were seeded in 48-well culture plates and incubated with test IgG (640 μg/ml) for 24 hours, and apoptosis in the harvested cells was measured with annexin V and 7-AAD (Apoptosis Detection Kit; BD Biosciences) with flow cytometry. Annexin V-positive/7-AAD-negative cells were measured as apoptotic cells.

### Statistical analysis

Data were analyzed by using the two-tailed Student *t *test or Mann-Whitney *U *test for continuous variables. Pairwise comparisons were assessed by using the Wilcoxon signed-rank test. Spearman rank correlation was used to explore the associations between anti-FLRT2 titer and clinical parameters. All analyses were performed by using Prism software (GraphPad Software, La Jolla, CA, USA). In all analyses, *P *< 0.05 was taken to indicate statistical significance.

## Results

### Detection of AECA activity with flow cytometry

**Figure 1:**
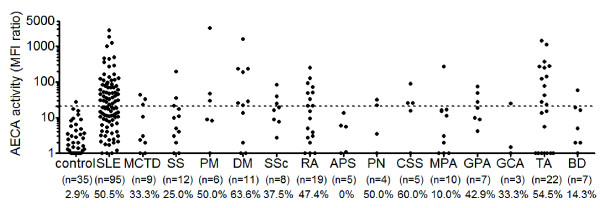
**Distribution of antiendothelial cell antibodies (AECAs)**. The distribution of AECAs in collagen diseases was measured with flow cytometry. Dots represent the data for individual subjects. The broken horizontal line indicates the cutoff value for high titers of AECAs (mean + 3 SD). Collagen diseases included systemic lupus erythematosus (SLE), mixed connective tissue disease (MCTD), Sjögren syndrome (SS), polymyositis (PM), dermatomyositis (DM), systemic sclerosis (SSc), rheumatoid arthritis (RA), antiphospholipid syndrome (APS), polyarteritis nodosa (PN), Churg-Strauss syndrome (CSS), microscopic polyangiitis (MPA), granulomatosis with polyangiitis (GPA), giant cell arteritis (GCA), Takayasu arteritis (TA), and Behçet disease (BD).

We first examined AECA activity in the sera from patients with collagen diseases by measuring binding activity of IgG to nonpermeabilized 7-AAD-negative HUVECs by using flow cytometry. The prevalence of AECAs was significantly higher in patients with SLE (50.5%) and other collagen diseases compared with normal controls (2.9%) (Figure 1). As these data indicated the presence of autoantigens on the EC surface, we constructed a retroviral vector system to identify cell-surface target molecules of AECAs with flow cytometry.

### Sorting of cells expressing cell-surface autoantigens with retroviral vector system

**Figure 2:**
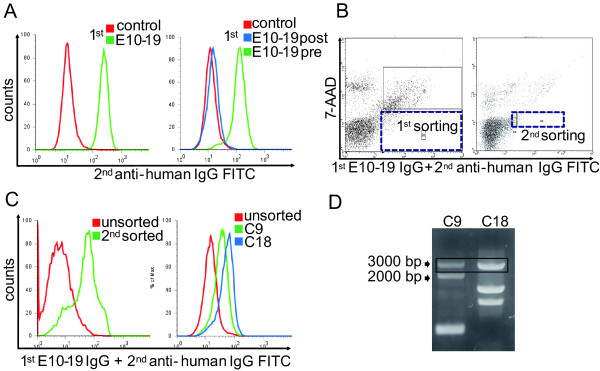
**Subcloning of autoantigen-expressing cells by using IgG from a patient with lupus nephritis**. **(A) **Nonpermeabilized HUVECs were stained with 1:10 diluted sera of control or E10-19 from a lupus nephritis patient (left), and 0.5 mg/ml of IgG of control or E10-19 collected before (pre) or after (post) the treatments (right) followed by secondary antibody and analyzed with flow cytometry. **(B) **HUVEC cDNA-expressing cells were stained with 0.5 mg/ml of E10-19 IgG followed by secondary antibody, and cells in the positive fraction were sorted (black dotted box). Left indicates first sorting, and right indicates second sorting. **(C) **Unsorted and second-sorted cells (left), and unsorted and two clones from second-sorted cells, C9 and C18, respectively (right), were stained with 0.5 mg/ml of E10-19 IgG followed by secondary antibody, and analyzed with flow cytometry. **(D) **HUVEC cDNA fragments inserted into the genomic DNA of C9 and C18 were amplified, and PCR products were electrophoresed on an 0.8% agarose gel.

Among sera with AECA activity, one sample (E10-19) from an SLE patient with active lupus nephritis (WHO IV) showed strong AECA activity (Figure 2A). We selected this serum sample as the prototype of AECAs for subsequent cell sorting. Purified IgG from E10-19 serum also showed strong binding to the surface of HUVECs, and IgG from the same patient collected after the treatment with 1 mg/kg prednisolone and intravenous cyclophosphamide showed remarkably reduced AECA activity (Figure 2A).

The YB2/0 cell line expressing HUVEC cDNA was generated by stable transfection of the HUVEC cDNA library with the retroviral vector system. After staining of this cell line with E10-19 IgG and FITC-conjugated secondary antibody, cells with strong FITC signals were sorted with flow cytometry. After cell expansion, we repeated one more round of cell sorting to concentrate E10-19 IgG-binding cells (Figure 2B). After the second sorting, cells bound to E10-19 IgG were markedly increased, and several cell clones were established from the E10-19 IgG-binding cell population by the limiting dilution method. Two distinct clones with different E10-19 IgG-binding activities and gene profiles of transfected HUVEC cDNA were established, C9 and C18 (Figure 2C and 2D).

### Identification of FLRT2 as a novel cell-surface autoantigen

**Figure 3:**
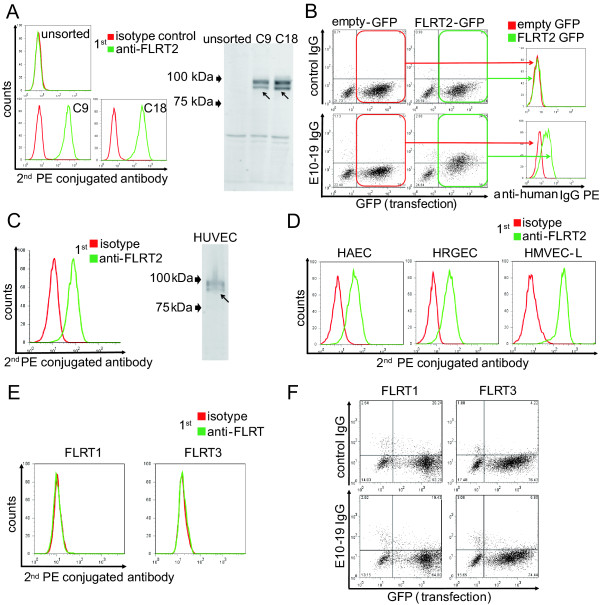
**Identification of FLRT2 as a novel autoantigen of AECAs**. **(A) **Unsorted, C9, and C18 were stained with isotype control or anti-FLRT2 antibody, followed by secondary antibody, and analyzed with flow cytometry (left). Western blotting of proteins from unsorted, C9, and C18 was performed, and they were stained with anti-FLRT2 antibody followed by secondary antibody (right). Arrows indicate the bands of FLRT2. Both of the two bands are FLRT2 because some of FLRT2 proteins were glycosylated. **(B) **Expression vector, empty-IRES-GFP, or FLRT2-IRES-GFP was transfected into HEK293T cells, and these cells were stained with 0.5 mg/ml of control IgG or E10-19 IgG, followed by secondary antibody, and analyzed with flow cytometry. Binding activities of IgG to cell-surface FLRT2 were analyzed in histograms (right) by gating for the GFP-positive transfected population (left). **(C) **HUVECs were stained with isotype control or anti-FLRT2 antibody followed by secondary antibody, and analyzed with flow cytometry (left). Western blotting of proteins from HUVECs was performed, and they were stained with anti-FLRT2 antibody followed by secondary antibody (right). The arrows indicate the bands of FLRT2. **(D) **HAECs, HRGECs, and HMVEC-Ls were stained with isotype control or anti-FLRT2 antibody followed by secondary antibody, and analyzed with flow cytometry. **(E) **HUVECs were stained with isotype control, anti-FLRT1 antibody, or anti-FLRT3 antibody followed by secondary antibody, and analyzed with flow cytometry. **(F) **Expression vector, FLRT1-IRES-GFP (left), or FLRT3-IRES-GFP (right) was transfected into HEK293T cells, and these cells were stained with 0.5 mg/ml of control IgG or E10-19 IgG, followed by secondary antibody, and analyzed with flow cytometry.

After PCR amplification and cloning of HUVEC cDNA inserted into the genomic DNA of C9 and C18, DNA sequencing was performed followed by BLAST analysis. PCR bands of around 3,000 bp in C9 and C18 (Figure 2D, black box) were found to be an identical gene, that is, fibronectin leucine-rich transmembrane protein 2 (*FLRT2*) cDNA (GenBank accession number NM_013231.4). Real-time quantitative PCR and microarray analysis of YB2/0, C9, and C18 also supported the conclusion that only the *FLRT2 *mRNA was overexpressed in both C9 and C18 (data not shown). Flow cytometry and Western blotting showed that FLRT2 protein was expressed on the cell surfaces of C9 and C18 (Figure 3A). Next, we generated an expression vector of FLRT2, which was transfected into HEK293T cells. E10-19 IgG showed significant binding activity to 7-AAD-negative FLRT2-expressing HEK293T cells (Figure 3B), indicating that E10-19 IgG has high anti-FLRT2 activity. Thus, the membrane protein FLRT2 was identified as a novel autoantigen.

Flow cytometry and Western blotting indicated that HUVECs and other ECs also expressed significant levels of FLRT2 on their cell surfaces (Figure 3C, D). FLRT2 is a member of the FLRT family, which includes FLRT1, FLRT2, and FLRT3 [38]. We examined whether other FLRTs were expressed on HUVECs with flow cytometry. Neither FLRT1 nor FLRT3 was expressed on the surface of these ECs, and E10-19 IgG showed no binding activity to either FLRT1 or FLRT3 (Figure 3E, F). These data indicated that among the FLRT family, FLRT2 was the only target molecule of AECAs.

### Inhibition test and epitope mapping

**Figure 4:**
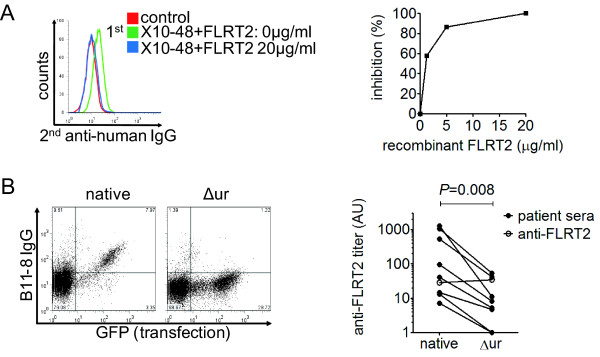
**Inhibition test and epitope mapping**. **(A) **Inhibition tests of binding activities to HUVECs were performed by using IgG from healthy donor (control) and anti-FLRT2 sera (X10-48) with soluble FLRT2 at the indicated concentrations. **(B) **Changes in binding activity to FLRT2 lacking the unique region (Δur) compared with native FLRT2 were analyzed by using anti-FLRT2 sera. Representative dot plot (left) and a summary of changes in each patient (right) are shown. Open circles show the binding activity to the intracellular FLRT2 domain.

We conducted inhibition tests to determine whether the AECA activities of anti-FLRT2-positive SLE patients were due to anti-FLRT2 activity. Incubation with soluble recombinant FLRT2 inhibited the binding of patient IgG to HUVECs (Figure 4A). We further investigated the epitope of anti-FLRT2 antibody. FLRT2 contains extracellular leucine-rich repeats, unique region, fibronectin type III domain, and a cytoplasmic tail. As mentioned earlier, FLRT2 was the only member of the FLRT family that was bound by SLE IgG, so we hypothesized that the unique region of FLRT2 may be the major epitope for anti-FLRT2 antibody. To investigate this hypothesis, an expression vector of FLRT2 lacking the unique region (FLRT2Δur) was generated. As shown in Figure 4B, the binding activity of the anti-FLRT2 antibody was significantly reduced when FLRT2 lacked its unique region (*P *= 0.008) compared with the equal binding activity of anti-FLRT2 antibody to the intracellular domain. These observations indicated that the major epitope was localized within the unique region of FLRT2.

### Distribution of patients with anti-FLRT2 activity

**Figure 5:**
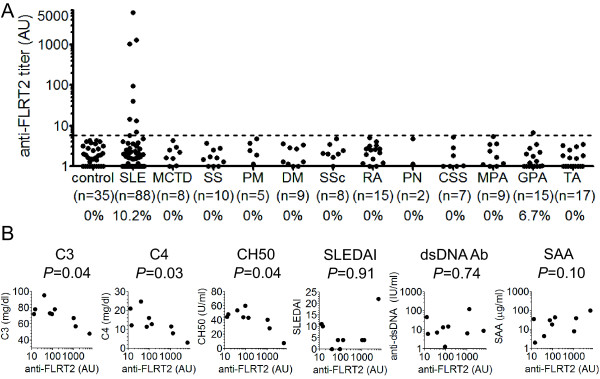
**Distribution of patients with anti-FLRT2 activity**. **(A) **The distribution of anti-FLRT2 activity in collagen diseases was measured with flow cytometry. Dots represent the data for individual subjects. The broken horizontal line indicates the cutoff value for high anti-FLRT2 activity (mean + 3SD). Collagen diseases included systemic lupus erythematosus (SLE), mixed connective tissue disease (MCTD), Sjögren syndrome (SS), polymyositis (PM), dermatomyositis (DM), systemic sclerosis (SSc), rheumatoid arthritis (RA), polyarteritis nodosa (PN), Churg-Strauss syndrome (CSS), microscopic polyangiitis (MPA), granulomatosis with polyangiitis (GPA), and Takayasu arteritis (TA). **(B) **Correlations of clinical parameters with anti-FLRT2 activity among anti-FLRT2-positive SLE patients are shown. CH50, 50% hemolytic complement activity; SLEDAI, SLE disease activity index; dsDNA Ab, anti-double-stranded DNA antibody; SAA, serum amyloid A.

Anti-FLRT2 activities were detected in nine (10.2%) of 88 patients with SLE and one (6.7%) of 15 patients with granulomatosis with polyangiitis (Wegener's). Healthy controls and other patients with collagen diseases, including diseases that showed a high prevalence of AECA activity, did not show anti-FLRT2 activity (Figure 5A). Strong anti-FLRT2 activities were detected in only SLE patients, indicating that anti-FLRT2 antibody is specific to SLE patients. Among 48 SLE patients with AECA positivity (Figure 1), 42 were examined for anti-FLRT2 activity, and nine patients (21.4%) were positive.

Among SLE patients with anti-FLRT2 positivity, anti-FLRT2 activity was significantly correlated with low levels of complement C3, C4, and CH50 (Figure 5B). No interrelations were found between anti-FLRT2 activity and the SLE disease activity index (SLEDAI), anti-dsDNA antibody titer, or serum amyloid A (SAA) level (Figure 5B).

### Induction of endothelial cell killing by CDC

**Figure 6:**
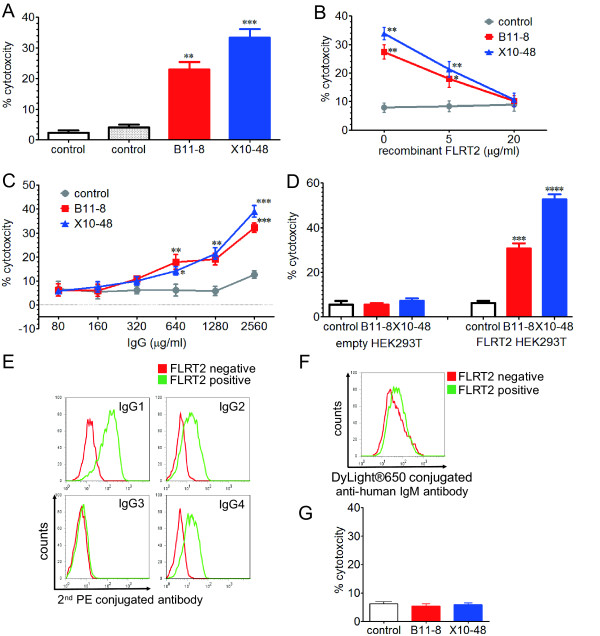
**Complement-dependent cytotoxicity (CDC) of anti-FLRT2 antibody**. CDC activities using two healthy control IgG and two anti-FLRT2 positive IgG, B11-8 and X10-48, at a concentration of 1.28 mg/ml with 1:3 diluted complement **(A)**, 1.28 mg/ml of IgG, and 1:3 diluted complement with recombinant FLRT2 at the indicated concentrations **(B)**, and various IgG concentrations with 1:6 diluted complement **(C) **against HUVECs were measured with the WST-1 assay. **(D) **CDC activities against mock transfected HEK293T cells (empty, left) and FLRT2-expressing HEK293T cells (FLRT2, right) by using 1.28 mg/ml of IgG and 1:3 diluted complement were measured with the WST-1 assay. HEK293T cells negative or positive for FLRT2 expression were stained with anti-FLRT2 antibody followed by secondary antibody against human IgG1, IgG2, IgG3, IgG4 **(E)**, and IgM **(F)**, and analyzed with flow cytometry. **(G) **ADCC activities using control IgG, B11-8, and X10-48, at a concentration of 1.28 mg/ml with an E:T ratio of 25:1 were determined with the lactate dehydrogenase detection method. Error bars indicate SD. *P < 0.05; **P < 0.01; ***P < 0.001; ****P < 0.0001.

We next assessed the functional significance of anti-FLRT2 antibody by using IgG from the sera of two SLE patients with high FLRT2 activity (B11-8 and X10-48). IgG with anti-FLRT2 activity showed significant CDC activity against HUVECs compared with IgG from normal controls (Figure 6A). This CDC activity was inhibited by incubation with soluble recombinant FLRT2, and increased with a higher concentration of IgG (Figure 6B, C). Strong CDC activity was induced against FLRT2-expressing HEK293T cells, but not against mock-transfected HEK293T cells (Figure 6D). These observations confirmed the ability of the anti-FLRT2 antibody to induce CDC activity by binding to cell-surface FLRT2.

We also analyzed the IgG subclasses of anti-FLRT2 antibody with flow cytometry. In all anti-FLRT2 active IgG subclasses, IgG1 and IgG2 activities were strong, and IgG3 was weak. The presence of IgG4 varied between patients (Figure 6E). Compared with IgG, weak IgM activity was detected (Figure 6F). None of these IgGs showed ADCC (Figure 6G).

### Other pathogenic roles as AECAs

**Figure 7:**
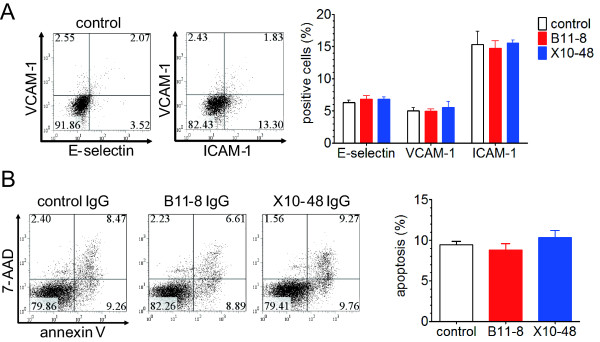
**Activation of HUVECs and induction of apoptosis**. **(A) **Expression of E-selectin, intercellular adhesion molecule 1 (ICAM-1), and vascular cell adhesion molecule 1 (VCAM-1) was analyzed with flow cytometry against HUVECs treated with 640 μg/ml of control and two anti-FLRT2-positive IgGs, B11-8 and X10-48. Representative graphs (left) and summarized graph (right) are shown. **(B) **HUVECs treated with control IgG and two anti-FLRT2-positive IgGs, B11-8 and X10-48, for 24 hours were stained with annexin V and 7-AAD and analyzed with flow cytometry. (Annexin V positive/7-AAD negative) cells were measured as apoptotic cells. Representative graphs (left) and summarized graph (right) are shown. Error bars indicate SD.

We examined further potentials for pathogenicity against EC activation and induction of apoptosis. The levels of expression of adhesion molecules (intercellular adhesion molecule 1 (ICAM-1), vascular cell adhesion molecule 1 (VCAM-1), and E-selectin) on HUVECs were not increased by incubation with IgG purified from B11-8 and X10-48 compared with control IgG (Figure 7A). Incubation of HUVECs with anti-FLRT2-positive IgG did not induce apoptosis (Figure 7B).

## Discussion

Although the existence of AECAs in patients with SLE and other collagen diseases has been reported, its pathogenic significance remains unknown. The localization of the AECA target molecule on the cell surface should be an important factor for its pathogenicity *in vivo*, in terms of accessibility of the target molecule to AECAs.

Our strategy to identify AECA target molecules is to use a retroviral vector system and flow cytometry. As the localization of cellular molecules depends on their structures, only cell-surface molecules are expressed on the surface of YB2/0 cells transfected with the HUVEC cDNA library. AECAs can bind only to cell-surface molecules in flow cytometry. Therefore, sorting of IgG-binding cells can concentrate and isolate cells expressing autoantigens (target molecules for AECAs) on the cell surface. Although this system may present difficulties in sorting cells at very low frequency, we isolated and cloned autoantigen-expressing cells by repeated sorting, and this system was shown to be useful to identify cell-surface autoantigens. Whereas some cell-surface molecules were identified with this system previously [39], this is the first report of autoantigen identification.

With purified IgG from one SLE patient with high AECA activity (E10-19), two distinct clones were isolated and established, both of which were shown to have an identical gene, *FLRT2*. As we confirmed that E10-19 IgG bound specifically to cell-surface FLRT2 and FLRT2 was expressed on the cell surface of ECs, we concluded that FLRT2 is a novel cell-surface autoantigen as a target molecule for AECAs in SLE patients.

Analysis of anti-FLRT2 activity among patients with various collagen diseases indicated that anti-FLRT2 antibody was specifically detected in SLE, and it accounted for 21.4% of cell-surface target molecules of AECAs in SLE. AECA activity of IgG from SLE patients with anti-FLRT2 activity was significantly inhibited by soluble recombinant FLRT2, indicating that FLRT2 is the major target on ECs for AECAs in these patients. Although heat-shock protein 60 (Hsp60) has been described as the target antigen of AECAs in SLE and has a proapoptotic effect [40,41], Hsp60 was not detected on freshly isolated unstressed HUVECs [40,41]. The remaining 78.6% of SLE patients with AECA activity in the present study may have other as-yet-unidentified target antigens.

FLRT2 is transmembrane protein and was identified as a novel gene family in the screening for extracellular matrix proteins expressed in muscle [38]. Although FLRT2 was shown to be expressed in the pancreas, skeletal muscle, brain, and heart with Northern blotting [38], we confirmed the expression of FLRT2 on HUVECs and other ECs (HAECs, HRGECs, and HMVEC-Ls), and treatment with neither tumor necrosis factor-α (TNF-α) nor lipopolysaccharide (LPS) induced the expression of FLRT2 (data not shown). E10-19 IgG did not bind to FLRT1 and FLRT3, and these two molecules were not expressed on ECs. Consistent with these findings, the major epitope for anti-FLRT2 antibody was localized in the unique region within the extracellular domain of FLRT2.

FLRT2 has been reported to modulate signaling, interact with fibroblast growth factor receptor, promote cell proliferation, participate in craniofacial development, and promote heart morphogenesis [42-46]. Although we hypothesized that anti-FLRT2 antibody may affect some cellular behavior and induce expression of adhesion molecules, cell proliferation, and apoptotic cell death without complement in ECs, we did not find these activities in the present study.

Among SLE patients with anti-FLRT2 activity, complement levels were correlated significantly with the anti-FLRT2 antibody titer. Moreover, anti-FLRT2 antibody induced cell damage in a complement-dependent manner, suggesting that it has pathogenic roles in immune-mediated vascular damage. CDC activity of AECAs was reported in patients with SLE, Takayasu arteritis, hemolytic-uremic syndrome, and Kawasaki disease [2,4,35,47,48]. Although ADCC activity was not proven in our study, similar observations of AECAs producing CDC but not ADCC were reported previously [35,48].

As demonstrated in this study, FLRT2 is widely distributed in various types of ECs. Therefore, it is possible that anti-FLRT2 antibody is linked to systemic vascular injury. These observations indicate that it is necessary to evaluate the contributions of anti-FLRT2 antibody to atherosclerotic lesions because chronic inflammation is atherogenic in SLE [49,50]. Administration of gammaglobulin was reported to reduce CDC of AECAs against ECs [35], and this may apply to anti-FLRT2 antibody-induced damage. Furthermore, incubation with soluble recombinant FLRT2 inhibited the AECA activity and CDC activity in patients with anti-FLRT2 positivity, which suggests that neutralizing anti-FLRT2 antibodies might be the specific therapeutic approach.

## Conclusions

We identified the membrane protein FLRT2 as a novel autoantigen of AECAs in SLE patients. Our retroviral vector system is useful for identification of cell-surface autoantigens. In addition to further investigations of the biologic significance of anti-FLRT2 antibody and its therapeutic applications, other cell-surface autoantigens of AECAs should be determined to achieve a comprehensive understanding of AECA-mediated vascular injury and the development of more-specific intervention strategies.

## Abbreviations

ADCC: antibody-dependent cell-mediated cytotoxicity; AECAs: anti-endothelial cell antibodies; APC: allophycocyanin; AUs: arbitrary units; CDC: complement-dependent cytotoxicity; DMEM: Dulbecco modified Eagle medium; ECs: endothelial cells; FBS: fetal bovine serum; FITC: fluorescein isothiocyanate; FLRT: fibronectin leucine-rich transmembrane protein; HAECs: human aortic endothelial cells; HMVEC-Ls: human lung microvascular endothelial cells; HRGECs: human renal glomerular endothelial cells; Hsp60: heat shock protein 60; HUVECs: human umbilical vein endothelial cells; ICAM-1: intercellular adhesion molecule 1; LPS: lipopolysaccharide; MFI: mean fluorescence intensity; PBS: phosphate-buffered saline; PCR: polymerase chain reaction; PE: phycoerythrin; RFUs: relative fluorescence units; SAA: serum amyloid A; SD: standard deviations; SDS: sodium dodecyl sulfate; SLE: systemic lupus erythematosus; SLEDAI: SLE disease activity index; TNF-α: tumor necrosis factor α; VCAM-1: vascular cell adhesion molecule 1; 7-AAD: 7-amino-actinomycin D.

## Competing interests

The authors declare that they have no competing interests.

## Authors' contributions

TS and HF carried out the molecular biologic studies, flow cytometry, clinical evaluation, and functional assays and drafted the manuscript. MO participated in the design of the study, performed the molecular biologic studies, and helped to draft the manuscript. KN, RW, YT, NT, and TI participated in its design and helped to draft the manuscript. HH conceived of the study, participated in its design and coordination, and helped to draft the manuscript. All authors read and approved the final manuscript.
